# The impact of eliminating within-country inequality in health coverage on maternal and child mortality: a Lives Saved Tool analysis

**DOI:** 10.1186/s12889-017-4737-2

**Published:** 2017-11-07

**Authors:** Adrienne Clermont

**Affiliations:** Department of International Health, Johns Hopkins Bloomberg School of Public Health, 615 N. Wolfe St., Baltimore, MD 21205 USA

**Keywords:** Equity, Inequality, Lives Saved Tool, MNCH, Intervention coverage

## Abstract

**Background:**

Inequality in healthcare across population groups in low-income countries is a growing topic of interest in global health. The Lives Saved Tool (LiST), which uses health intervention coverage to model maternal, neonatal, and child health outcomes such as mortality rates, can be used to analyze the impact of within-country inequality.

**Methods:**

Data from nationally representative household surveys (98 surveys conducted between 1998 and 2014), disaggregated by wealth quintile, were used to create a LiST analysis that models the impact of scaling up health intervention coverage for the entire country from the national average to the rate of the top wealth quintile (richest 20% of the population). Interventions for which household survey data are available were used as proxies for other interventions that are not measured in surveys, based on co-delivery of intervention packages.

**Results:**

For the 98 countries included in the analysis, 24–32% of child deaths (including 34–47% of neonatal deaths and 16–19% of post-neonatal deaths) could be prevented by scaling up national coverage of key health interventions to the level of the top wealth quintile. On average, the interventions with most unequal coverage rates across wealth quintiles were those related to childbirth in health facilities and to water and sanitation infrastructure; the most equally distributed were those delivered through community-based mass campaigns, such as vaccines, vitamin A supplementation, and bednet distribution.

**Conclusions:**

LiST is a powerful tool for exploring the policy and programmatic implications of within-country inequality in low-income, high-mortality-burden countries. An “Equity Tool” app has been developed within the software to make this type of analysis easily accessible to users.

**Electronic supplementary material:**

The online version of this article (10.1186/s12889-017-4737-2) contains supplementary material, which is available to authorized users.

## Background

Over the past two decades, the global health community’s attention has shifted beyond the obvious disparities between high-income and low-income countries to the issue of within-country inequality among low-income countries [1]. Access to healthcare, and thus health outcomes, is often very different for the richest and poorest people in a single country. This disparity can be measured in many different ways, perhaps the simplest of which is the difference in intervention coverage across income groups [2, 3].

An important policy and program planning tool for examining the link between intervention coverage and health outcomes is the Lives Saved Tool (LiST) modeling software [4]. LiST includes a range of best-practice community and facility-based health interventions in maternal, neonatal, and child health (MNCH) and nutrition, and is used to model the impact of scaling up intervention coverage on mortality and other key health outcomes. LiST can be used to examine equity questions. It has been used by groups such as Save the Children [5, 6] and UNICEF [7] to analyze how within-country coverage disparities affect MNCH and advocate for reduction of inequalities.

This purpose of this paper is to document the methodology for using LiST to explore inequality. First, it will describe the more intensive approach to creating LiST projections, using as an example an analysis that models the impact of eliminating within-country disparities in health coverage using household survey data. Second, it will present the recently developed “Equity Tool,” which provides a simple and automated way for LiST users to explore the mortality impact of inequality in the countries for which data exists.

## Methods

LiST creates projections for individual countries that include preloaded country-specific demographic and health status data. This includes mortality rates, cause-of-death structure, disease burden, nutritional status, and current intervention coverage levels, so that a LiST projection takes into account the health context of each country in its calculations. Users can then scale up intervention coverage rates over a time period of their choice, and display change in mortality rates over time or number of deaths averted (“lives saved”) relative to the baseline year as results. The impact of a given intervention is determined by the baseline number of cause-specific deaths, the change in coverage level, the effectiveness of the intervention, and the affected fraction (the proportion of the target population susceptible to treatment).

One intervention scale-up scenario that can be modeled, of interest for the question at hand, is increasing coverage from the national average (the default in the baseline year) to the level of the top wealth quintile (WQ), or richest 20% of the population, in each country. The analysis presented here used nationally representative household survey data from Demographic and Health Surveys (DHS) and Multiple Indicator Cluster Surveys (MICS) for 98 countries [8, 9]. The list of countries included in this analysis, as well as the year of the most recent survey data available for this analysis and the current under-five mortality rate, is available in Additional file 1.

Coverage levels for each health intervention included in LiST were disaggregated by wealth quintile to create an “inequality ratio” (the top WQ coverage rate divided by the national coverage rate). The ratio was set to 1 in the case where the top WQ had a lower coverage rate than the national average, in order to avoid decreasing coverage of these interventions in the scale-up scenario. The ratio was then applied to the latest national coverage data for each country in a LiST projection, using the national rate for the year 2016 and the top wealth quintile rate (national rate * inequality ratio) for the year 2017. It was then possible to calculate the number of lives saved in 2017 (relative to 2016) due to the increase in intervention coverage, as well as the change in mortality rates.

Due to limitations in what is measured through household surveys, only 18 direct coverage measures are currently available in LiST (Table 1). Based on research and expert consensus, LiST makes certain default assumptions about how measured coverage of certain simple and easily measured interactions with the healthcare system (particularly antenatal care visits and childbirth in a health facility) are translated into more detailed and complex individual components of MNCH care (for example, syphilis detection and treatment during antenatal care, or neonatal resuscitation during childbirth). These assumptions are documented in Additional file 2.

**Table 1:** LiST interventions scaled up in analysis, with proxies

Intervention	Included in household surveys?	Proxy (Backup)
Contraceptive prevalence rate (CPR)	Yes	
Antenatal care^a^	Yes	
Tetanus toxoid vaccination		Antenatal care
Intermittent preventive treatment of malaria in pregnancy (IPTp)	Yes	(Antenatal care)
Syphilis detection and treatment		Antenatal care
Iron supplementation in pregnancy		Antenatal care
Hypertensive disorder case management		Antenatal care
MgSO_4_ management of pre-eclampsia		Antenatal care
Skilled birth attendant^a^	Yes	
Facility delivery^a^	Yes	
Clean birth practices		Facility delivery
Immediate assessment and stimulation		Facility delivery
Labor and delivery management		Facility delivery
Neonatal resuscitation		Facility delivery
Antenatal corticosteroids for preterm labor		Facility delivery
Antibiotics for pPRoM		Facility delivery
MgSO_4_ management of eclampsia		Facility delivery
Active management of the third stage of labor (AMSTL)		Facility delivery
Breastfeeding promotion		Facility delivery
Clean postnatal practices	Yes	(Facility delivery)
Vitamin A supplementation	Yes	
Improved water source	Yes	
Water connection in the home	Yes	(Improved sanitation)
Improved sanitation	Yes	(Water connection in the home)
Hand washing with soap		Hygienic disposal of children’s stools
Hygienic disposal of children’s stools	Yes	(Improved sanitation)
Household ownership of insecticide treated bednet (ITN)	Yes	
DPT vaccine	Yes	
H. influenzae b vaccine	Yes	(DPT vaccine)
HepB vaccine		DPT vaccine
Pneumococcal vaccine		DPT vaccine
Rotavirus vaccine		DPT vaccine
Measles vaccine	Yes	
Thermal care for neonatal prematurity		Facility delivery
Injectable antibiotics neonatal sepsis/pneumonia		Facility delivery
Oral rehydration solution (ORS)	Yes	(Oral antibiotics for pneumonia)
Antibiotics for treatment of dysentery		ORS
Zinc for treatment of diarrhea		ORS
Oral antibiotics for pneumonia	Yes	(ORS)
Vitamin A for treatment of measles		Vitamin A for prevention
Artemisinin-based combination therapies (ACTs) for malaria	Yes	

^a^These measures do not have a direct impact in LiST, but rather are used to determine coverage of the specific interventions that they are made up of (also listed in the table)

Certain additional assumptions were made specifically for this analysis in order to create as complete a picture as possible of within-country inequality, despite incomplete data availability. Based on which interventions are co-delivered and thus likely to have similar levels of inequality in coverage, the inequality ratios for interventions where data are available were used as proxies for other interventions where measurements are not available (or as backups in cases where specific surveys did not have the relevant data available). As a result, a total of 38 interventions included in LiST were scaled up for the “full” version of this analysis (Table 1). In order to understand the impact of these assumptions on the results, a “limited” analysis was also carried out, in which only interventions that had survey data available were scaled up in each country.

Contraceptive prevalence was only scaled up in countries were the baseline fertility rate was above the replacement rate (2.33 children per woman). The average inequality ratio over all 98 countries, for interventions where data is directly available in household surveys (not including proxies), is shown in Table 2.

**Table 2:** Inequality ratios for interventions available in household surveys

Intervention	Average ratio
Water connection in the home	2.332
Improved sanitation	1.787
Artemisinin-based combination therapies (ACTs) for malaria	1.746
Health facility delivery	1.475
Skilled birth attendant	1.397
Hygienic disposal of children’s stools	1.356
Clean postnatal practices	1.354
Antenatal care	1.318
Contraceptive prevalence rate (CPR)	1.310
Intermittent preventive treatment of malaria in pregnancy (IPTp)	1.291
Oral antibiotics for pneumonia	1.237
Improved water source	1.222
Oral rehydration solution (ORS)	1.165
H. influenzae b vaccine	1.153
DPT vaccine	1.131
Vitamin A supplementation	1.122
Measles vaccine	1.106
Household ownership of insecticide treated bednet (ITN)	0.945

The inequality ratio is calculated by dividing the top wealth quintile coverage rate by the national coverage rate

## Results

In the baseline scenario with no intervention scale-up, there were a total of 5,379,309 under-five deaths in 2017. In the scale-up scenario, the number of under-five deaths was reduced to 3,675,397 in 2017, meaning that 32% of under-five deaths were prevented through the elimination of within-country inequality.

The impact of reducing inequality was even greater for neonatal deaths: There were 2,416,983 neonatal deaths in the baseline scenario and 1,274,459 neonatal deaths in the scale-up scenario in 2017, meaning that 47% of neonatal deaths were prevented.

The impact was smaller for post-neonatal deaths: There were 2,962,326 post-neonatal deaths in the baseline scenario and 2,400,938 post-neonatal deaths in the scale-up scenario in 2017, meaning that 19% of post-neonatal deaths were prevented.

**Fig. 1:**
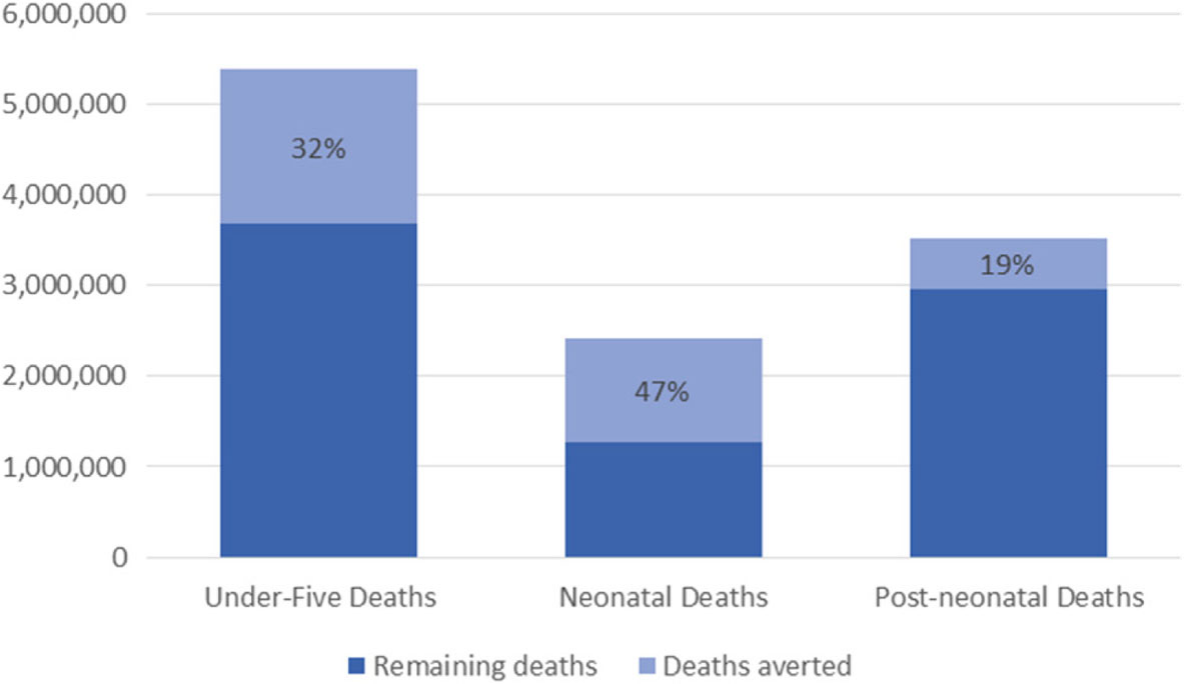
Percent of deaths averted through equity scale-up

**Fig. 2:**
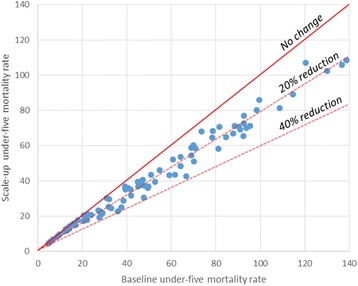
Change in under-five mortality rate

**Fig. 3:**
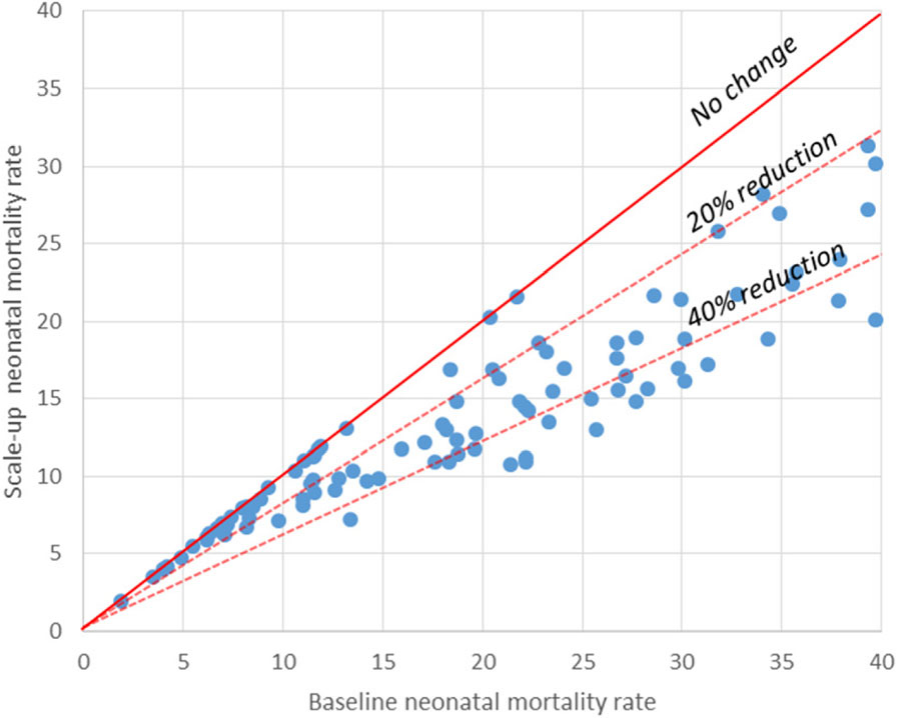
Change in neonatal mortality rate

Figure 1 displays these results. Figure 2 shows the decrease in under-five mortality rates relative to baseline for each individual country, and Fig. 3 shows the decrease in neonatal mortality rates relative to baseline for each individual country.

The “limited” analysis, which only scaled up interventions for which survey data was available, produced somewhat more conservative estimates: 24% of under-five deaths, 34% of neonatal deaths, and 16% of post-neonatal deaths were prevented. The results of the limited analysis are presented in more detail in Additional file 3. This provides a more conservative lower bound for estimating the impact of reducing within-country inequality on child survival.

LiST is also able to attribute lives saved to specific interventions, without double-counting when multiple interventions are scaled up simultaneously [4]. The number of lives saved takes into account not just interventions’ inequality ratios, but also their baseline coverage level and their effectiveness on decreasing child mortality. In this analysis, the most important interventions in terms of number of child lives saved were labor and delivery management (225,644 lives saved), water connection in the home (104,517 lives saved), case management of neonatal infection (101,846 lives saved), oral antibiotics for pneumonia (96,470 lives saved), neonatal resuscitation (77,886 lives saved), and improved sanitation (60,625 lives saved). Of these, only case management of neonatal infection was not included in the “limited” analysis.

## Discussion

For the 98 countries included in the analysis, between a quarter and a third of child deaths and between a third and a half of neonatal deaths could be prevented by scaling up national coverage of key health interventions to the level already enjoyed by the wealthiest 20% of that country’s population.

The ratios in Table 2 present some interesting but intuitive trends regarding the relative inequality levels of different healthcare interventions [10]. On average, the most unequal interventions across all countries were those related to childbirth (skilled birth attendant and health facility delivery) and water and sanitation indicators (water connection in the home and improved sanitation). This is unsurprising, as these interventions require significant infrastructure investments, in terms of health facilities and sanitation infrastructure, respectively. Access to these facilities tends to be highly unequal, and is not as easily remedied as other non-infrastructure interventions. The interventions with the highest levels of inequality also corresponded to the highest number of lives saved in the analysis, indicating that these are also highly effective interventions for improving child survival.

The most equal (or most “pro-poor”) interventions were those that are typically distributed via community-based mass campaigns and only require a single, cheap commodity – vaccines, vitamin A supplementation, and insecticide treated bednets (ITNs). In fact, ITNs were the only intervention to have an average equity ratio of less than 1 (meaning the top wealth quintile has a lower coverage rate than the national average), which is likely indicative of the fact that mass bednet distribution campaigns in many countries in recent years have successfully targeted poorer, more rural households, where risk of malaria is greater. This is confirmed by the literature, which has previously identified ITNs as one of the most equitable interventions, at least in countries with rapidly increasing coverage [11].

There are some limitations to this analysis, particularly related to data availability. For those countries where coverage data were several years old, levels of inequality were assumed to have remained constant in the intervening period (i.e., that the inequality ratios could still be applied in 2016). If inequality has significantly decreased since the survey was conducted, this analysis may overestimate the number of deaths due to within-country inequality. Excluding the four countries with survey data prior to 2005 did not change the results of the analysis.

In addition, assumptions were made about which inequality ratios to apply for interventions where survey data were unavailable. These assumptions are thought to be reasonable, in that they are based on co-delivery of intervention packages; for example, components of neonatal care that are delivered in health facilities immediately after birth (not measured in household surveys) could reasonably be assumed to have a similar coverage differential to that of health facility childbirth (measured in surveys). However, in order to address the additional uncertainty that these assumptions introduce, a second “limited” analysis was also conducted to provide a more conservative estimate. Although these values are lower than the “full” analysis, they nonetheless demonstrate a significant impact of eliminating within-country disparities in health interventions.

### LiST equity tool

Given the increased interest in equity issues, the LiST development team has sought to make this analysis easily accessible for users of the software. LiST now includes an “Equity Tool” app that automatically calculates a simple analysis for every country with coverage survey data available; this provides a rapid way for users to explore the impact of scaling up individual interventions to the top WQ coverage rate in the country of their choice.

**Fig. 4:**
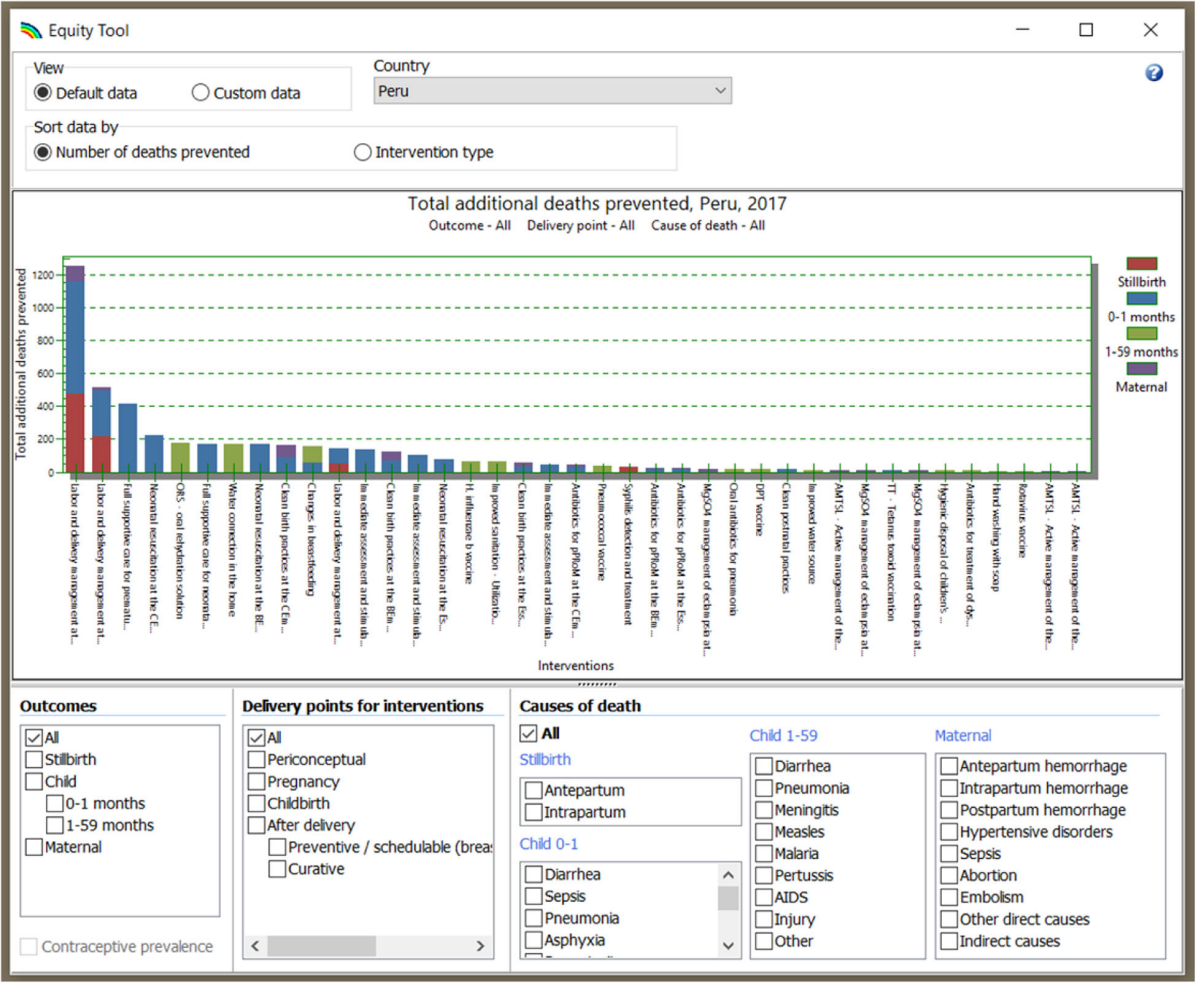
Equity tool in the LiST software

Figure 4 shows an image of the Equity Tool. The methodology behind this tool entails individually scaling up each intervention in the software from the national average to the top WQ level (using the inequality ratio approach described above), and counting the number of deaths averted by this scale-up. This is done for each intervention for which there is data, and the results are then displayed in a graph. Interventions are ranked in order of the number of lives saved through scale-up, so the interventions with the largest impact (due to the largest level of inequality in baseline coverage and the highest effectiveness) are at the left. Different colors represent stillbirths, neonatal deaths, child deaths, and maternal deaths. The checkboxes at the bottom of the screen allow for disaggregation of the data by specific population groups, intervention types, or causes of death.

As this tool allows users to see only the impact of scaling up one intervention at a time, it is meant as a quick “first glance” at inequality in a given country. Users may then use the full LiST software to further explore the impact of scaling up packages up multiple interventions.

## Conclusions

The discrepancies in healthcare coverage between the top WQ and the rest of the country “seem to be unfair and avoidable” [10], and are thus worthy of note and of policy intervention where possible. In particular, top WQ coverage rates are a useful advocacy tool because they represent a level of intervention coverage that presumably cannot be rejected as infeasible in the local context – given that 20% of the country’s population is already there. As Braveman and Tarimo observed almost 15 years ago, “improvements in health for privileged groups should suggest what could, with political will, be possible for all” [1].

Measurement of within-country inequality is complex; there are many dimensions of inequality that could be assessed instead of wealth (e.g., education, gender, urban/rural, religion, or ethnicity) [11]. In addition, household asset index measures of wealth have their shortcomings, and how they are computed can affect subsequent wealth-based inequality measures [12]. There may be other, more nuanced ways of representing within-country inequality than simply comparing coverage between wealth quintiles, but other studies have shown this simplified approach to be reasonable [2, 3].

LiST does not directly capture the impact of factors outside of the health sector, such as mother’s education or household income. Instead, changes in these factors (over time, or between wealth quintiles) are assumed to translate into changes in health intervention coverage. These issues are important – a recent study suggests that half of child-mortality reduction since 1990 can be attributed to investments outside the health sector [13] – but this does not invalidate the LiST approach. Previous research using LiST in Bangladesh found that modeled mortality rates for each of the five wealth quintiles, based on changes in intervention coverage across quintiles, corresponded closely with quintile-specific neonatal and post-neonatal mortality rates as measured by the DHS [14]. The present analysis suggests that despite the many other advantages that the wealthiest populations enjoy, simply ensuring that all citizens have equal access to healthcare can have a significant impact on child survival.

Although global improvements in health intervention coverage have included more rapid improvements among disadvantaged groups over the last 10 years, resulting in a narrowed inequality gap over time [11], this analysis shows that significant disparities persist. As a result, data collection and analysis disaggregated by wealth quintile, or by other measures believed to correlate with disadvantages in access to healthcare, must remain a priority for researchers and policymakers. LiST can help to produce program planning and advocacy messages around this issue.

## Additional files

Additional file 1: List of countries included in analysis. (PDF 92 kb)

Additional file 2: List of the default assumptions in LiST related to antenatal care and childbirth. (PDF 138 kb)

Additional file 3: “Limited” inequality analysis results. (PDF 181 kb)

## Abbreviations

DHS: Demographic and Health Surveys
ITN: Insecticide treated bednet
LiST: Lives Saved Tool
MICS: Multiple Indicator Cluster Surveys
MNCH: Maternal, neonatal, and child health
WQ: Wealth quintile
