# Supplementary material for: The impact of eliminating within-country inequality in health coverage on maternal and child mortality: a Lives Saved Tool analysis
Source: BMC Public Health. 2017 Nov 7;17(Suppl 4):734. doi: 10.1186/s12889-017-4737-2 (PMC5688502; doi:10.1186/s12889-017-4737-2)
Supplement: Supplementary file 1 — List of countries included in analysis. (PDF 92 kb) [file 12889_2017_4737_MOESM1_ESM.pdf]

Additional File 1

Countries included in analysis

Country	Most recent survey with inequality data	Under-five mortality rate (2016)
Afghanistan	2010	91.1
Albania	2008	14.0
Argentina	2011	12.5
Armenia	2010	14.1
Azerbaijan	2006	31.7
Bangladesh	2011	37.6
Barbados	2012	13.0
Belarus	2012	4.6
Belize	2011	16.5
Benin	2011	99.5
Bhutan	2010	32.91
Bolivia	2008	38.4
Bosnia and Herzegovina	2011	5.4
Brazil	2006	16.4
Burkina Faso	2010	88.6
Burundi	2010	81.7
Cambodia	2010	28.7
Cameroon	2011	87.9
CAR	2010	130.1
Chad	2010	138.7
Colombia	2010	15.9
Comoros	2012	73.6
Congo Brazzaville	2011	45.0
Congo Democratic Republic	2013	98.3
Costa Rica	2011	9.7
Cote d'Ivoire	2011	92.6
Dominican Republic	2013	30.9
Egypt	2008	24.0
Ethiopia	2011	59.2
Gabon	2012	50.8
Gambia	2013	68.9
Georgia	2005	11.9
Ghana	2011	61.6
Guatemala	2008	29.1
Guinea	2012	93.7
Guinea Bissau	2006	92.5
Guyana	2009	39.4
Haiti	2012	69.0
Honduras	2011	20.4
India	2005	47.7
Indonesia	2012	27.2
Iraq	2011	32.0
Jamaica	2011	15.7
Jordan	2012	17.9
Kazakhstan	2010	14.1
Kenya	2008	49.4
Kyrgyzstan	2012	21.3
Laos	2011	66.7

Lesotho	2009	90.2
Liberia	2013	69.9
Macedonia	2011	5.5
Madagascar	2008	49.6
Malawi	2010	64.0
Maldives	2009	8.6
Mali	2012	114.7
Mauritania	2011	84.7
Moldova	2012	15.8
Mongolia	2010	22.4
Morocco	2003	27.6
Mozambique	2011	78.5
Namibia	2013	45.4
Nepal	2011	35.8
Nicaragua	2001	22.1
Niger	2012	95.5
Nigeria	2013	108.8
Palestine	2010	21.1
Pakistan	2012	81.1
Peru	2012	16.9
Philippines	2013	28.0
Rwanda	2010	41.7
Sao Tome and Principe	2008	47.3
Senegal	2014	47.2
Serbia	2014	6.7
Sierra Leone	2013	120.4
Somalia	2006	136.8
South Africa	1998	40.5
South Sudan	2010	92.6
St. Lucia	2012	14.3
Sudan	2010	70.1
Suriname	2010	21.3
Swaziland	2010	60.7
Syria	2006	12.9
Tajikistan	2012	44.8
Tanzania	2010	48.7
Thailand	2005	12.3
Timor Leste	2009	52.6
Togo	2013	78.4
Trinidad and Tobago	2006	20.4
Tunisia	2011	14.0
Turkey	2003	13.5
Uganda	2011	54.6
Ukraine	2012	9.0
Uzbekistan	2006	39.1
Vanuatu	2007	27.5
Vietnam	2010	21.7
Yemen	2006	41.9
Zambia	2007	64.0
Zimbabwe	2014	70.7
