# Supplementary material for: The impact of eliminating within-country inequality in health coverage on maternal and child mortality: a Lives Saved Tool analysis
Source: BMC Public Health. 2017 Nov 7;17(Suppl 4):734. doi: 10.1186/s12889-017-4737-2 (PMC5688502; doi:10.1186/s12889-017-4737-2)

Additional File 2

Default assumptions in LiST related to antenatal care and childbirth

Measures of antenatal care coverage and childbirth in a health facility are available from nationally representative household surveys. These measures in and of themselves do not have an effect on child survival in LiST. Instead, based on research and expert consensus, LiST makes certain assumptions about coverage of their more specific component parts (each of which has an effect size based on the scientific research literature).

Antenatal care

Antenatal care in LiST is defined as the percent of women attending four or more antenatal visits during pregnancy (ANC4+). Data about coverage of specific components of antenatal care are not available in household surveys. As a result, LiST makes the following assumptions about coverage of component interventions:

ANC component	Coverage assumption in LiST
Syphilis detection and treatment	Coverage varies based on level of antenatal care coverage:<br>• If ANC4+ is less than 40%, then this indicator will equal 20% of ANC4+<br>• If ANC4+ is between 40% and 75%, then this indicator will equal 50% of ANC4+<br>• If ANC4+ is between 75% and 95%, then this indicator will equal 70% of ANC4+<br>• If ANC4+ is 95% or greater, then this indicator will equal 100% of ANC4+
Hypertensive disorders case management	Assumed to be 5% of ANC4+ coverage at all levels
Diabetes case management	Assumed to be 5% of ANC4+ coverage at all levels
Malaria case management	Assumed to be 5% of ANC4+ coverage at all levels
MgSO4 management of pre-eclampsia	Assumed to be 5% of ANC4+ coverage at all levels

Other components of antenatal care included in LiST are tetanus toxoid vaccine, iron supplementation, and intermittent preventive treatment of malaria (IPTp), but these are not linked to antenatal care because coverage data for these components are typically available from household surveys.

Childbirth

Data on the percent of babies delivered in a health facility are available from household surveys; however, further detail on the level of health facility or the components of childbirth care that are present are not available. As a result, LiST makes the following assumptions about distribution of facility deliveries (FacilDeliv) across three levels of health facilities:

Facility type	Definition / signal functions	Coverage assumption in LiST
Essential Care	Deliveries at this level of care are assumed to be in facilities that include monitoring of labor progress with a partograph, detection of complications, infection control via a clean delivery, and episiotomy if needed. For the neonate, this includes routine care practices including immediate drying, skin-to-skin contact or immediate wrapping for thermal care, and clean cord cutting.<br><br>In LiST, the default assumption is that all Essential Care facilities provide clean birth practices, immediate assessment and stimulation of the newborn, labor and delivery management, and neonatal resuscitation.	The percentage of deliveries at this level are calculated according to the following formula:<br>• If FacilDeliv is less than 30%, then Essential care deliveries are 90% of FacilDeliv<br>• If FacilDeliv is between 30% and 50%, then Essential care deliveries are 50% of FacilDeliv<br>• If FacilDeliv is between 50% and 95%, then Essential care deliveries are 25% of FacilDeliv<br>• If FacilDeliv are 95% or greater, then Essential care deliveries are 0% of FacilDeliv
Basic Emergency Obstetric Care (BEmOC) facility	Deliveries at this level of care are assumed to be in facilities that meet the WHO's guidelines for Basic Emergency Obstetric and Newborn Care. BEmOC facilities must be able to perform seven signal functions: administer parenteral antibiotics; administer parenteral anticonvulsants; administer parenteral oxtyocics; manual removal of placenta; removal of retained products (manual vacuum aspiration); assisted vaginal delivery (with vacuum extractor or forceps); and neonatal resuscitation with bag and mask.<br><br>In LiST, the default assumption is that all BEmOC facilities provide clean birth practices, immediate assessment and stimulation of the newborn, labor and delivery management, neonatal resuscitation, antibiotics for pPRoM,	The percentage of deliveries at this level are calculated according to the following formula:<br>• If FacilDeliv is less than 30%, then BEmOC deliveries are 0% of FacilDeliv<br>• If FacilDeliv is between 30% and 50%, then BEmOC deliveries are 30% of FacilDeliv<br>• If FacilDeliv is between 50% and 95%, then BEmOC deliveries are 15% of FacilDeliv<br>• If FacilDeliv are 95% or greater, then BEmOC deliveries are 0% of FacilDeliv

	MgSO4 management of eclampsia, and active management of the third stage of labor (AMTSL).	
Comprehensive Emergency Obstetric Care (CEmOC) facility	Deliveries at this level of care are assumed to be in facilities that meet the WHO's guidelines for Comprehensive Emergency Obstetric and Newborn Care (CEmOC). CEmOC facilities must be able to perform the seven signal functions of BEmOC, plus surgery (e.g. Caesarean section) and blood transfusion.<br><br>In LiST, the default assumption is that all CEmOC facilities provide clean birth practices, immediate assessment and stimulation of the newborn, labor and delivery management, neonatal resuscitation, antibiotics for pPRoM, MgSO4 management of eclampsia, active management of the third stage of labor (AMTSL), and induction of labor for pregnancies lasting 41+ weeks.	The percentage of deliveries at this level are calculated from Health Facility Deliveries (FacilDeliv) according to the following formula:<br>• If FacilDeliv is less than 30%, then CEmOC deliveries are 10% of FacilDeliv<br>• If FacilDeliv is between 30% and 50%, then CEmOC deliveries are 20% of FacilDeliv<br>• If FacilDeliv is between 50% and 95%, then CEmOC deliveries are 60% of FacilDeliv<br>• If FacilDeliv are 95% or greater, then CEmOC deliveries are 100% of FacilDeliv

The distribution algorithm described in the table above is summarized as follows:

Figure A1.1: Distribution of births by health facility type in LiST

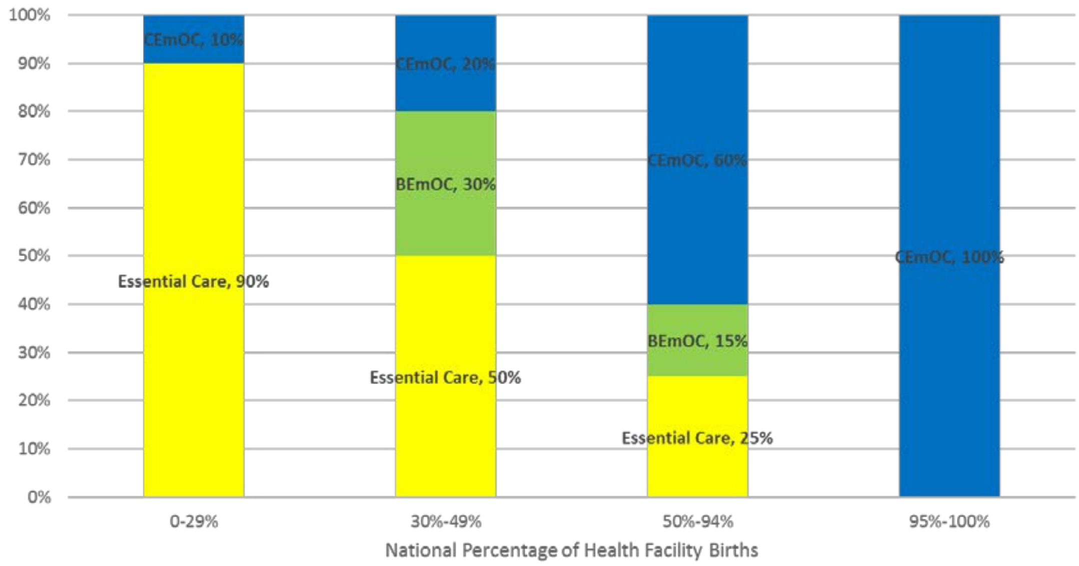

Supplement: Supplementary file 2 — List of the default assumptions in LiST related to antenatal care and childbirth. (PDF 138 kb) [file 12889_2017_4737_MOESM2_ESM.pdf]
