# Supplementary material for: The impact of eliminating within-country inequality in health coverage on maternal and child mortality: a Lives Saved Tool analysis
Source: BMC Public Health. 2017 Nov 7;17(Suppl 4):734. doi: 10.1186/s12889-017-4737-2 (PMC5688502; doi:10.1186/s12889-017-4737-2)

Additional File 3

Results from “limited” inequality analysis

In the baseline scenario with no intervention scale-up, there were a total of 5,379,309 under-five deaths in 2017. In the scale-up scenario, the number of under-five deaths was reduced to 4,086,556 in 2017, meaning that 24% of under-five deaths were prevented through the elimination of within-country inequality.

The impact of reducing inequality was even greater for neonatal deaths: There were 2,416,983 neonatal deaths in the baseline scenario and 1,605,059 neonatal deaths in the scale-up scenario in 2017, meaning that 34% of neonatal deaths were prevented.

The impact was smaller for post-neonatal deaths: There were 2,962,326 post-neonatal deaths in the baseline scenario and 2,481,497 post-neonatal deaths in the scale-up scenario in 2017, meaning that 16% of post-neonatal deaths were prevented.

Figure A2.1: Percent of deaths averted through equity scale-up (limited)

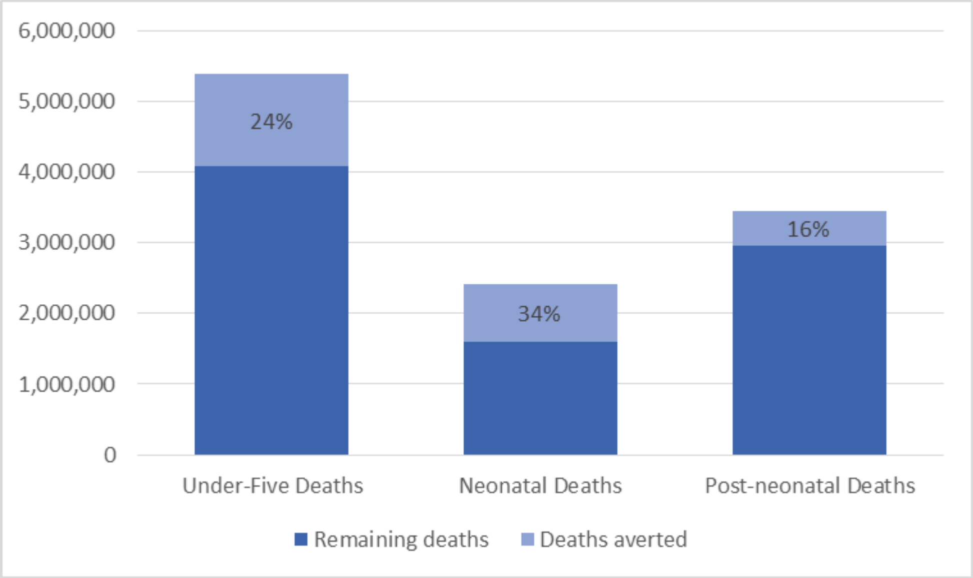

Figure A2.2: Change in under-five mortality rate (limited)

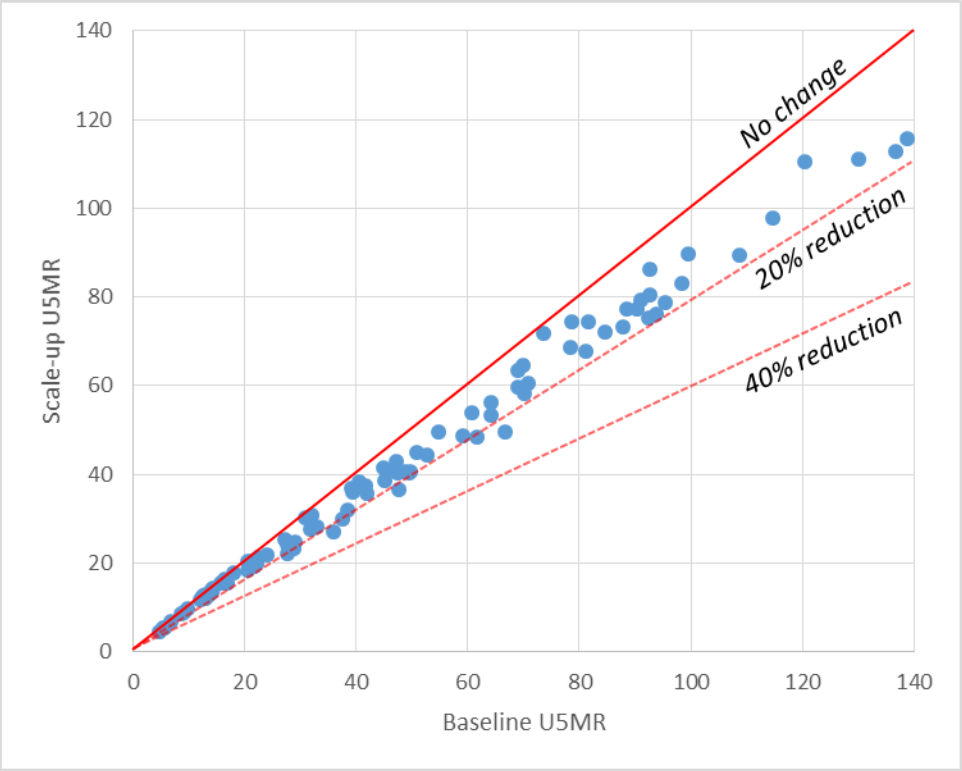

Figure A2.3: Change in neonatal mortality rate (limited)

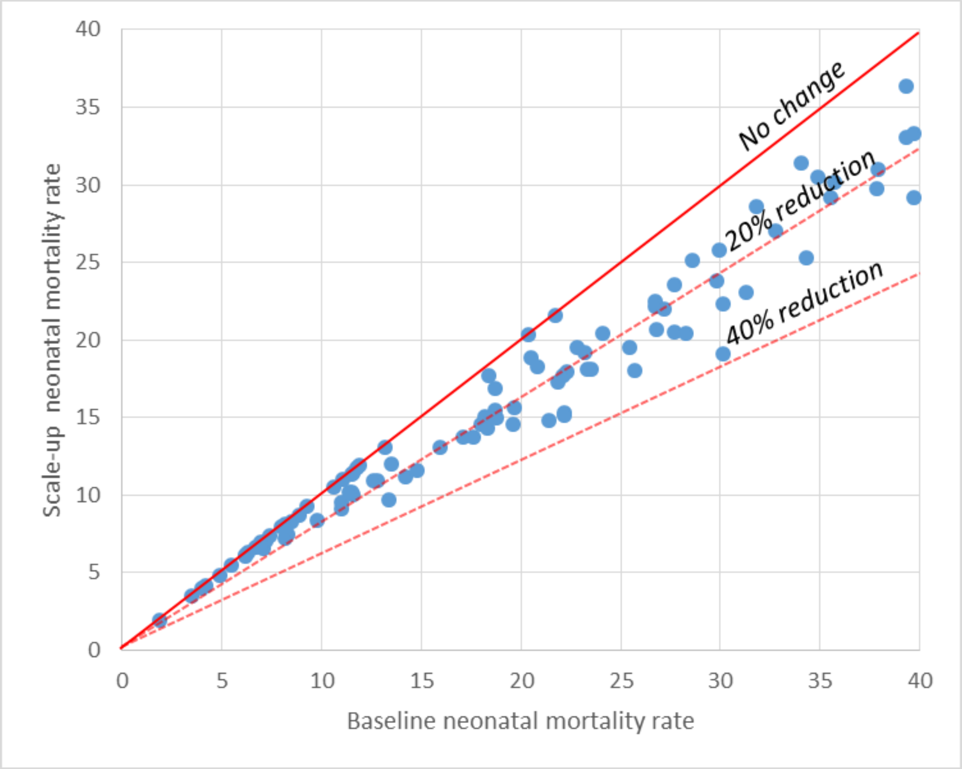

Supplement: Supplementary file 3 — “Limited” inequality analysis results. (PDF 181 kb) [file 12889_2017_4737_MOESM3_ESM.pdf]
